# A Pedagogical-Interindividual Model of Situated Critical Thinking in Higher Education

**DOI:** 10.3390/jintelligence14100233

**Published:** 2026-10-01

**Authors:** Maritza Palma-Luengo, Carlos Ossa-Cornejo, Nelly Lagos-San Martín, Sofía Carrasco Palma

**Affiliations:** 1 Department of Educational Sciences, University of Bío-Bío, Chillán 3780000, Chile; cossa@ubiobio.cl (C.O.-C.); nlagos@ubiobio.cl (N.L.-S.M.); 2 Faculty of Medicine, Universidad San Sebastián, Concepción 4080871, Chile; scarrascop4@correo.uss.cl

**Keywords:** critical thinking, higher education, instructional mediation, metacognition, motivation, socio-linguistic interaction

## Abstract

Critical thinking is an essential competency in higher education, particularly in contexts characterized by complexity, uncertainty, information overload, and technological transformation. However, various models explain critical thinking primarily from cognitive perspectives, thereby limiting understanding of the conditions that foster its activation, regulation, and advancement in authentic educational settings. This article proposes a pedagogical-interindividual model of situated critical thinking in higher education, developed through a conceptual review of theoretical and empirical literature regarding critical thinking, metacognition, motivation, social interaction, language, and instructional mediation. The model is organized as a relational and functional architecture rather than as a linear sequence and distinguishes interconnected components according to their respective functions: teacher instructional mediation as an instructional-contextual condition; argumentative socio-linguistic interaction as an interindividual mechanism; motivation and metacognition as individual factors of activation and regulation; critical cognitive processes as the operational core; situated critical thinking as performance; and evidence-based decision-making as an educational outcome. The model hypothesizes that situated critical thinking may emerge from direct and indirect relationships among students’ internal resources, discursive practices, and intentionally designed pedagogical conditions.

## 1. Introduction

Critical Thinking (CT) has been recognized as a fundamental competency for twenty-first-century higher education, responding to the emerging educational demands of the contemporary world (Anderson & Beach, 2022). Its development is particularly relevant for future professionals who will be required to address situations characterized by uncertainty, information overload, diversity of perspectives, and the need to make evidence-based decisions. In this regard, CT is considered an essential competency for lifelong learning, employability, responsible professional practice, and active civic engagement (Thornhill-Miller et al., 2023).

From an intraindividual perspective, CT has traditionally been defined as a set of higher-order cognitive processes associated with analysis, inference, evaluation, argumentation, and reasoning directed toward evidence-based decision-making (Facione, 1990; Halpern, 2014). This approach has been fundamental to the development of the field because it has enabled the identification of its core components and the development of models and instruments for its assessment. However, an understanding focused exclusively on cognition is insufficient to explain why students who possess reasoning skills mobilize their knowledge inconsistently when confronted with complex academic tasks. This highlights the need to consider factors that influence the activation, regulation, and maintenance of CT, as well as the pedagogical conditions under which these skills are put into practice.

From an interindividual perspective, CT not only involves the student’s internal processes but is also understood as a situated competency constructed through interaction among individuals, tasks, instructors, peers, content, language, and the sociocultural context (C. J. Ossa-Cornejo & Díaz-Mujica, 2017; Davies & Barnett, 2015). Accordingly, dialogue, argumentation, counter argumentation, negotiation of meaning, and the use of academic language enable individuals to externalize, contrast, and reconstruct reasoning. Therefore, the depth of CT achieved in university classrooms also depends on pedagogical opportunities that foster active intellectual participation, shared reflection, and situated argument construction.

From an educational perspective, this approach poses significant challenges for universities, which bear the responsibility of generating educational processes aimed at analysing information, evaluating evidence, contrasting perspectives, constructing well-founded arguments, and making reasoned decisions in response to complex situations (Facione, 1990; Halpern, 2014; Davies & Barnett, 2015). Consequently, including CT merely as a general statement within graduate profiles is insufficient. Rather, it should be established as an explicit, planned, and assessable learning outcome throughout the educational trajectory (Ennis, 2018; Thompson, 2011). Achieving this requires intentional, progressive, and contextualized learning experiences that promote deep reasoning through authentic problems, argumentative discussion, inquiry, formative feedback, and evidence evaluation (Abrami et al., 2015; Bezanilla et al., 2019; Ostendorf & Thoma, 2022).

Within this framework, teacher instructional mediation assumes a central role as an instructional-contextual condition that organizes opportunities for CT development. Specifically, strategies such as problematizing content, posing higher-order questions, facilitating argumentative discussions, providing formative feedback, conducting case analyses, and assigning tasks that require justification of decisions based on evidence can activate motivation toward deep thinking and promote metacognitive processes of planning, monitoring, and evaluating one’s own reasoning (Schraw & Dennison, 1994; Eccles & Wigfield, 2002; Halpern, 2014; Rodríguez-Sandoval et al., 2022; Ostendorf & Thoma, 2022). In this way, instructional mediation enables students to mobilize their cognitive skills consciously, strategically, and purposefully in authentic academic situations.

Despite these advances, much of the existing research continues to address CT from partial perspectives, focusing primarily on cognitive skills (Angelelli et al., 2023; Ellerton, 2022), motivational factors associated with the disposition to think critically (Valenzuela & Nieto, 2008; Valenzuela et al., 2014, 2023; Berestova et al., 2022), or metacognitive processes related to the regulation of thinking (Rivas et al., 2022). Only recently have some studies begun to explore the relationships among cognitive, metacognitive, and motivational factors associated with CT in an integrated manner (C. J. Ossa-Cornejo et al., 2023; Castro-Cáceres et al., 2025). This fragmentation limits understanding of the mechanisms through which CT is activated, regulated, expressed, and deepened in specific university contexts. Furthermore, in the Latin American context, there remains a limited availability of validated instruments and models that simultaneously consider these relationships among university students (C. J. Ossa-Cornejo et al., 2017, 2018).

In response to this scenario, this article proposes a pedagogical-interindividual model of situated CT in higher education. The model recognizes that critical cognitive processes constitute the operational core of CT; that metacognition functions as a regulatory system; that motivation serves as a condition that activates and sustains deep thinking; that social interaction and language operate as socio-linguistic mechanisms for the externalization and reconstruction of reasoning; and that teacher instructional mediation constitutes a pedagogical condition that enables the emergence and deepening of CT in the university classroom. In this way, the proposal seeks to integrate intraindividual and interindividual perspectives on CT, avoiding excessive expansion of the construct while simultaneously providing a situated explanation of its development in higher education.

## 2. Method: Conceptual Review and Synthesis

**Figure 1:**
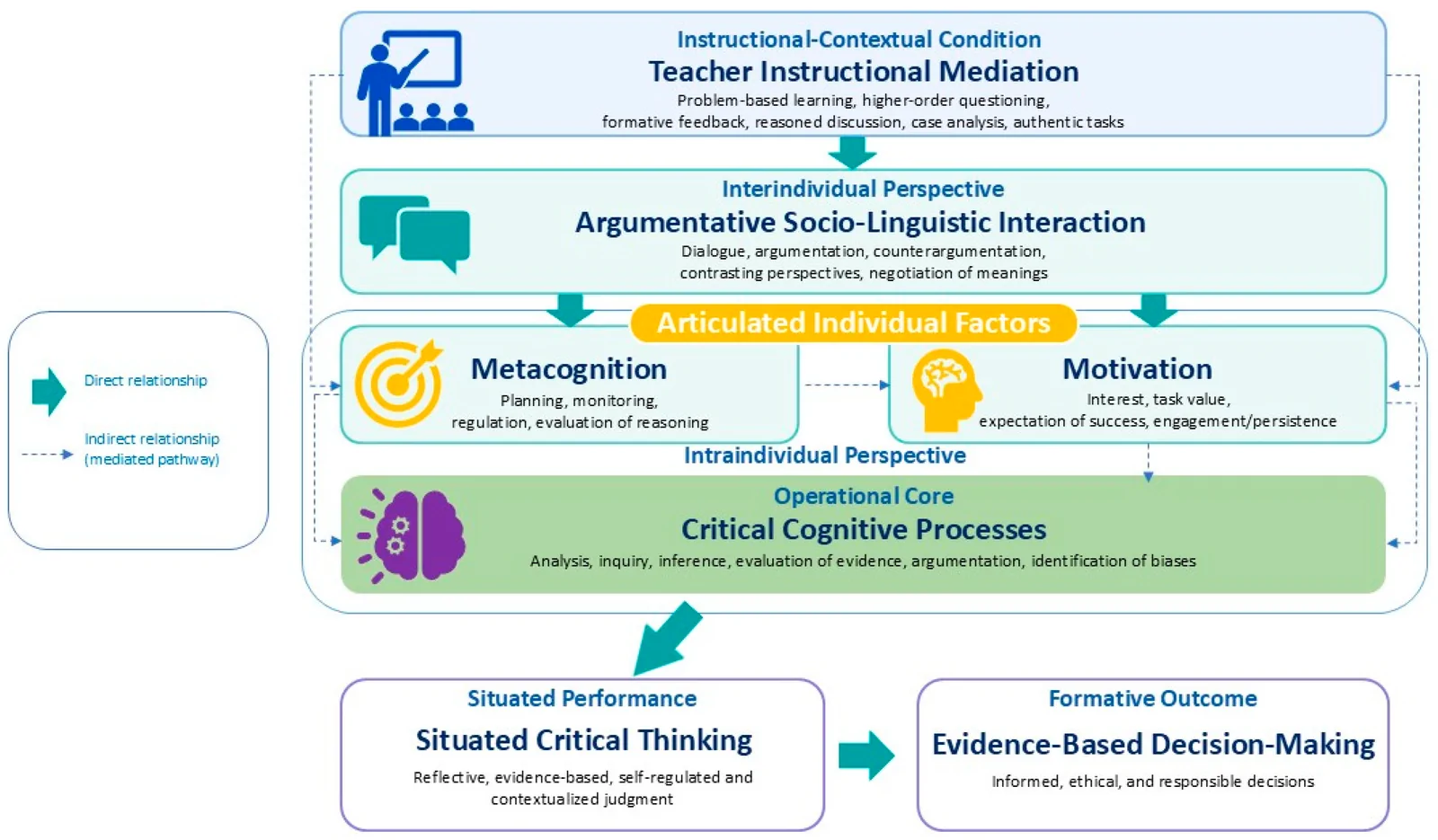
Proposed pedagogical-interindividual model of Critical Thinking in higher education. (Source: Authors’ own elaboration).

This manuscript is presented as a Hypothesis Paper because its main purpose is to develop a theoretically grounded and empirically testable model of critical thinking in higher education. The conceptual review was used as a theory-building procedure to identify the main components involved in critical thinking, assign their functional roles within the model, and formulate the directional relationships represented in Figure 1 and in the proposed propositions.

The conceptual synthesis organizes previously studied components into a functional architecture that explains how critical thinking may be activated, regulated, socially mediated, and expressed in situated decision-making. Therefore, the contribution of this paper lies in the formulation of theoretically derived propositions intended to guide future empirical validation through quantitative, qualitative, or mixed-methods research.

The conceptual synthesis drew on sources identified through Web of Science, Scopus, ERIC, SciELO, and Google Scholar, with searches conducted between May and June 2026 and covering classical and recent contributions published between 1979 and 2026 on critical thinking, metacognition, motivation, reasoning, socio-linguistic interaction, instructional mediation, artificial intelligence, and higher education.

The selection of sources was guided by four criteria: (a) classical and widely recognized contributions to the conceptualization and assessment of critical thinking; (b) empirical and theoretical studies addressing cognitive, metacognitive, motivational, socio-linguistic, and instructional dimensions; (c) research situated in higher education contexts; and (d) Latin American and Chilean evidence relevant to contextualizing the proposal. These criteria allowed the reviewed literature to be organized according to its conceptual relevance, theoretical consistency, and contribution to understanding critical thinking from both intraindividual and interindividual perspectives.

The analytical process followed an integrative logic in three stages. First, intraindividual approaches, focused on cognitive skills, metacognition, and motivation, were distinguished from interindividual approaches associated with social interaction, language, and pedagogical mediation. Second, the function of each component within the model was delineated, differentiating constitutive CT processes, regulatory factors, activating conditions, socio-linguistic mechanisms, and instructional conditions. Third, these relationships were articulated into a conceptual model that explains CT as a situated, regulated, motivated, socially mediated, and didactically promoted performance.

To this end, future research should advance toward empirical studies capable of validating the proposed model across different higher education contexts. Such studies should combine multidimensional instruments with performance-based tasks in order to assess not only students’ self-reported perceptions, but also their actual critical thinking performance in authentic academic situations. Likewise, classroom observation and the analysis of discursive interaction would make it possible to examine how teacher mediation, socio-linguistic interaction, argumentation, and feedback contribute to the activation and regulation of critical thinking processes.

## 3. Theoretical Foundations of Critical Thinking

The theoretical foundations of the proposed model are organized according to the functional levels represented in Figure 1. First, the intraindividual components of CT are presented, including the cognitive core and the regulatory and activating roles of metacognition and motivation. Second, socio-linguistic and argumentative interaction is addressed as an interindividual mechanism through which critical reasoning is externalized, contrasted, and reconstructed. Third, teacher instructional mediation is examined as an instructional-contextual condition that organizes opportunities for the development of CT in higher education. Finally, situated critical thinking performance and evidence-based decision-making are presented as the performance and outcome components of the model.

From the perspective of cognitive psychology, thinking is understood as a set of complex mental processes that enable individuals to interpret reality, construct knowledge, solve problems, and make decisions (Sternberg, 1985; Flavell, 1979). Within this framework, it is important to distinguish between thinking and reasoning. Thinking refers to a broad cognitive activity through which individuals process information, develop mental representations, and generate meaning; reasoning, by contrast, constitutes a more structured form of thinking aimed at establishing relationships among premises, analysing evidence, drawing inferences, and constructing well-founded conclusions (Holyoak & Morrison, 2005; Leighton & Sternberg, 2004). In this sense, CT can be understood as a higher-order mode of thinking that mobilizes reasoning processes to analyze information, evaluate evidence, question assumptions, and formulate judgments that guide problem solving and evidence-based decision-making.

Over recent decades, CT has become established as a key competency for contemporary society (Dekker, 2020; Moreno-Pinado & Velázquez-Tejeda, 2017), particularly in contexts characterized by complexity, uncertainty, technological transformation, and the massive circulation of information (Anggraeni et al., 2023; Milagros-Baloa & Navas-Alvario, 2024; Thornhill-Miller et al., 2023). Consequently, CT is no longer conceived solely as a skill linked to logical reasoning but rather as a cross-cutting competency associated with lifelong learning, professional performance, civic participation, and responsible decision-making (Ennis, 2011; Saíz & Rivas, 2012). In this context, international organizations such as UNESCO (2022), the Organisation for Economic Co-operation and Development (OECD, 2015), and the World Economic Forum (2016) have highlighted its relevance as an essential twenty-first-century competency.

In higher education, CT has been recognized as a relevant predictor of academic achievement and professional performance (Bezanilla et al., 2021; Butler et al., 2017; Ren et al., 2020). Its development enables students to question assumptions, critically evaluate information sources, recognize biases, and engage with social, scientific, and academic discourses from a more autonomous and rigorous standpoint (C. Ossa-Cornejo et al., 2020). Thus, CT transcends the strictly academic domain and emerges as a competency necessary for responsible professional practice and ethical participation in complex and digitalized societies (Facione, 2015; Halpern, 2014; Elder & Paul, 2019; UNESCO, 2022). Nevertheless, despite its importance, debates persist regarding its nature, components, and the conditions that foster its development in specific educational contexts.

### 3.1. Intraindividual Components of Critical Thinking

From an intraindividual perspective, CT has been primarily understood as a set of cognitive skills and internal dispositions associated with analysis, inference, evaluation, argumentation, and evidence-based decision-making (Facione, 1990; Ennis, 2011; Halpern, 2014; Paul & Elder, 2019). This viewpoint, widely developed within cognitive psychology, has been essential for delimiting the cognitive core of the construct and guiding its assessment by identifying the mental processes that enable individuals to reason, evaluate evidence, and formulate justified judgments. However, although this approach clarifies which skills are involved in CT, it is insufficient to explain how those skills are activated, regulated, and deepened in authentic university learning situations.

In this regard, C. J. Ossa-Cornejo and Díaz-Mujica (2017) propose understanding CT from a social praxis perspective, distinguishing between an intraindividual and an interindividual approach. The former conceives CT as a set of mental processes through which individuals analyse, evaluate, infer, and decide. The latter understands CT as a situated and socially mediated practice constructed through interaction with others, tasks, discourses, cultural norms, and educational contexts. From this interindividual perspective, cognitive processes are articulated with social, motivational, cultural, linguistic, and pedagogical factors that condition how CT is activated, expressed, and given meaning in specific educational contexts.

Therefore, this article assumes that critical cognitive processes constitute the operational core of CT, but that its development in higher education also requires consideration of the factors and conditions that allow these processes to be activated, regulated, expressed, and deepened. This distinction makes it possible to articulate a pedagogical-interindividual understanding of CT in which cognition is recognized as the constitutive foundation, metacognition as the regulatory system, motivation as the activating and sustaining condition, socio-linguistic interaction as an interindividual mechanism, and teacher instructional mediation as an instructional-contextual condition.

#### 3.1.1. Cognitive Core: Critical Cognitive Processes

Although CT has been one of the most extensively studied constructs in recent decades, there is still no single universally accepted definition. One of the most influential conceptualizations emerged from the Delphi Project of the American Philosophical Association, led by Facione (1990), which defines CT as a deliberate and self-regulated process of judgment involving the interpretation, analysis, evaluation, and inference of relevant information for the formulation of conclusions and evidence-based decision-making. Similarly, Ennis (1985) conceptualizes CT as reflective and reasonable thinking focused on deciding what to believe or what to do, emphasizing its intentional and action-oriented character. Paul and Elder (2003) later expanded this perspective by highlighting the importance of applying intellectual standards such as clarity, precision, accuracy, relevance, depth, and logic in evaluating the quality of reasoning. Halpern (1998, 2014), for her part, emphasizes the transfer of CT skills to problem solving and decision-making in real-life situations, underscoring their applicability beyond academic contexts.

Franco and Almeida (2017) argue that CT constitutes a complex intellectual competency that enables individuals to analyse information, evaluate evidence, compare alternatives, and formulate reasoned judgments in response to diverse problems. These authors stress that this competency should be understood as a cross-cutting capacity for professional and civic development whose growth requires educational experiences that foster reasoning, argumentation, and problem-solving in authentic contexts. Consequently, the acquisition of disciplinary knowledge also involves the mobilization of cognitive skills aimed at understanding, interpreting, and acting in a well-founded manner when confronted with complex situations.

Likewise, Saíz and Rivas (2008, 2011) conceive CT as a trainable competency that integrates reasoning, argumentation, decision-making, and problem-solving processes. Their proposal emphasizes the systematic development of skills that enable the evaluation of evidence, identification of biases, generation of alternatives, and justification of conclusions through rational criteria. Their intervention programs also recognize that critical reasoning training can be strengthened through self-regulation processes and motivational strategies that promote engagement with cognitively demanding tasks, particularly through the ARDESOS program and subsequent developments.

In the Chilean context, Miranda (2003) conceptualizes CT as a cognitive competency oriented toward questioning, problematizing, and critically examining knowledge and truths assumed to be unquestionable. From an operational perspective, this competency is expressed through three fundamental skills: analysis, inquiry, and communication. The analytical dimension includes hypothesis formulation, evidence evaluation, and problem-solving processes; the inquiry dimension relates to the systematic search, selection, and evaluation of relevant information; and the communicative dimension refers to the ability to organize, argue, and disseminate ideas through various discursive forms. This proposal is particularly relevant for higher education because it provides a cognitive structure that helps explain how students mobilize reasoning processes to address complex problems and justify their decisions.

These approaches converge in understanding CT as a set of complex cognitive skills oriented toward analysis, evaluation, argumentation, and evidence-based decision-making. They also recognize that CT involves the capacity to use knowledge flexibly in order to solve problems, evaluate evidence, and construct reasoned judgments across diverse contexts. Nevertheless, despite acknowledging the complexity of the construct, most of these approaches continue to privilege cognitive skills as the principal explanation for its development and performance.

This tendency is reflected in recent literature reviews, such as Bubikova-Moan et al. (2026), who identify sustained growth in CT research within higher education and initial teacher education, while also noting persistent conceptual and methodological difficulties related to defining the construct, the diversity of definitions employed, and the limited consideration of non-cognitive factors. Similarly, Franco and Almeida (2017) argue that CT development depends not only on the acquisition of knowledge and reasoning skills but also on students’ ability to apply these skills in authentic and contextually meaningful situations.

Taken together, these approaches delineate an intraindividual perspective in which CT possesses a clearly identifiable cognitive core associated with reasoning, problem-solving, and the construction of well-founded judgments. However, this perspective provides only a partial explanation of the conditions that enable such skills to be effectively activated, regulated, and sustained in specific educational contexts. Students may possess the cognitive resources required for critical reasoning, yet without motivational engagement, metacognitive regulation, dialogic opportunities, and instructional mediation, those resources may remain underused or inconsistently expressed.

#### 3.1.2. Metacognition as a Regulatory Factor

Metacognition is understood in the proposed model as an intraindividual regulatory factor that enables students to plan, monitor, regulate, and evaluate their own reasoning. From this perspective, metacognition does not replace the cognitive core of CT; rather, it regulates the way in which critical cognitive processes are mobilized during complex academic tasks. The incorporation of opportunities for planning, monitoring, self-assessment, and revision of one’s own reasoning promotes the metacognitive regulation of learning, as proposed in the work of Flavell (1979), Schraw and Dennison (1994), Ku and Ho (2010), Magno (2010), Fleur et al. (2021), Paul and Elder (2019), and Halpern (2014).

Within higher education, metacognitive regulation is especially relevant because students must frequently confront open-ended problems, ambiguous information, conflicting sources, and tasks that require justification of decisions. In these contexts, the quality of CT depends not only on the possession of analytical or evaluative skills, but also on the capacity to observe one’s own thinking, identify errors or biases, adjust strategies, and evaluate the strength of the arguments produced. Therefore, metacognition functions as a regulatory system that supports the conscious, strategic, and reflective use of critical cognitive processes.

However, the role of metacognition should not be interpreted as an empirically established causal pathway within the proposed model. Rather, it is advanced as a theoretically grounded relationship that requires future empirical validation. Current evidence suggests relevant associations between metacognition, motivation, and CT. In addition, recent Latin American evidence has examined metacognitive strategies as predictors of critical thinking in higher education contexts (Vigo-Ingar et al., 2025). However, the specific ordering among these components remains an open question that should be examined through longitudinal, multilevel, and intervention-based designs. 

#### 3.1.3. Motivation as an Activating and Sustaining Factor

Motivation is understood in this model as an intraindividual activating and sustaining factor of CT. Its function is to promote students’ willingness to engage in cognitively demanding tasks, persist in the face of complexity, assign value to reasoning, and sustain the intellectual effort required to analyse, evaluate, argue, and make well-founded decisions. From the Expectancy-Value framework, Eccles and Wigfield (2002) argue that student engagement depends on expectations of success and the value attributed to a task. Consistent with this perspective, Valenzuela and Nieto (2008), Olivares et al. (2013) and Valenzuela et al. (2014, 2023) maintain that the activation of critical thinking skills requires motivation to use them, particularly when tasks demand effort, persistence, and rigorous evaluation of information.

Recent empirical evidence across different undergraduate programs supports the relationship between motivation and CT, although it may vary according to disciplinary area and the type of instrument used. In university Psychology students, Valenzuela et al. (2023) found that the motivational factors associated with the Expectancy–Value model explained between 8% and 17% of the variance in CT performance, assessed by means of the PENCRISAL test and the CCTST (California Critical Thinking Skills Test), with expectancy of success standing out as one of the components with the greatest predictive capacity. In initial teacher education, C. J. Ossa-Cornejo et al. (2023), in a sample of 435 education students from specialties such as mathematics, history, science, special education, and early childhood education, demonstrated through structural equation modeling a positive and significant relationship between metacognition and motivation to think critically, as well as a positive, though weaker, relationship between motivation and CT skills. These findings suggest that metacognition may contribute indirectly to critical thinking through its positive association with motivation to think critically.

In the field of Nursing, Berger et al. (2021) found among Chilean students that motivation for CT was associated with academic factors such as Problem-Based Learning, motivation fostered by instructors, and the explicit incorporation of CT within disciplinary courses. Similarly, AlOtaibi et al. (2023), studying nursing students in Saudi Arabia and Egypt, identified positive correlations between motivation toward CT and cognitive-processing strategies in mental health and psychiatry courses. Berestova et al. (2022), working with university students in Russia and Bulgaria, found that certain types of academic motivation, including self-development, cognition, achievement, and self-esteem, were associated with better performance on the Watson-Glaser Critical Thinking Appraisal. Finally, Berger et al. (2024), in students enrolled in Engineering, Economics and Business Sciences, Nursing, and Rehabilitation programs, observed disciplinary differences in the components of motivation for CT, particularly regarding expectations of success. This finding suggests that the relationship between motivation and CT may be conditioned by the educational cultures and curricular experiences characteristic of each academic field.

Taken together, this body of evidence supports the view that motivation is not an ancillary component but rather a relevant condition for activating and sustaining the use of critical thinking skills across diverse university contexts. However, within the proposed pedagogical-interindividual model, its effect should not be interpreted in isolation or as a linear relationship; instead, it should be understood in conjunction with metacognition, critical cognitive processes, and the instructional conditions that provide meaning, challenge, and purpose to academic tasks. Thus, motivation may operate as an activating and potentially moderating factor of CT insofar as it promotes students’ persistence when confronted with tasks that require deep analysis, regulation of thought, and evidence-based decision-making.

### 3.2. Interindividual Mechanism: Socio-Linguistic and Argumentative Interaction

From the interindividual perspective, social interaction and language are understood in this model as a socio-linguistic mechanism that mediates critical thinking, rather than as independent dimensions of the construct. Language constitutes the means through which individuals organize, express, justify, question, and reconstruct meaning within contexts of social interaction. Therefore, critical thinking is not limited to the analysis, evaluation, or logical inference of information; it is also expressed and developed through discursive practices oriented toward argumentation, the comparison of perspectives, and the collaborative construction of knowledge (Facione, 1990; Ennis, 2011; Paul & Elder, 2019).

From a sociocultural perspective, Vygotsky (1981, as cited in Rozo Poveda & Calvache López, 2023) argued that the development of higher psychological functions occurs through social interaction mediated by language. Drawing on Bakhtin’s dialogic perspective, Sisto (2015) argues that knowledge is configured dialogically through the confrontation and articulation of multiple voices. These contributions make it possible to understand that language functions not only as a means of communication but also as a cognitive and social tool that enables individuals to externalize reasoning, subject it to review, and reconstruct it through dialogue.

Within educational settings, communicative interactions foster processes of metacognitive reflection by enabling students to examine their assumptions, identify biases, justify their positions, and critically evaluate both their own ideas and those of others within dialogic and argumentative approaches to critical thinking (Mercer, 2000; Felton, 2004, Kuhn, 2018, 2019). Along the same lines, Lipman (2003) and Bezanilla et al. (2018) emphasize that dialogue, deliberation, and the competent use of language strengthen democratic participation, coexistence, and evidence-based decision-making.

Accordingly, socio-linguistic and argumentative interaction is proposed as a theoretical mechanism that may mediate the relationship between instructional conditions and the mobilization of critical cognitive processes. This mediation should be understood as a theoretically derived expectation rather than as an empirically established mechanism. Its validation requires future research capable of analysing the quality of classroom discourse, the nature of argumentative exchanges, and the ways in which students use evidence, counterarguments, and justification in situated academic practices (Nussbaum et al., 2023).

### 3.3. Instructional-Contextual Condition: Teacher Mediation

Teacher instructional mediation constitutes an instructional-contextual condition that organizes opportunities for the development of CT in the university classroom. From this perspective, CT does not develop solely through exposure to declarative knowledge, but through learning experiences that engage students with authentic problems, intellectual challenges, open-ended questions, and situations that require them to analyze evidence, compare perspectives, formulate arguments, and justify decisions (Halpern, 2014; Paul & Elder, 2019; Rodríguez-Sandoval et al., 2022).

Specifically, strategies such as problematizing content, posing higher-order questions, facilitating argumentative discussions, providing formative feedback, conducting case analyses, and assigning tasks that require justification of decisions based on evidence can activate motivation toward deep thinking and promote metacognitive processes of planning, monitoring, and evaluating one’s own reasoning (Schraw & Dennison, 1994; Eccles & Wigfield, 2002; Halpern, 2014; Abrami et al., 2015; Bezanilla et al., 2019; Rodríguez-Sandoval et al., 2022; Ostendorf & Thoma, 2022). In this way, instructional mediation enables students to mobilize their cognitive skills consciously, strategically, and purposefully in authentic academic situations.

From this perspective, pedagogical mediation fulfills a central function because it organizes the conditions that enable students to move from prior conceptions toward more complex forms of reasoning through thinking routines, scaffolding, feedback, and progressive activities that foster intellectual autonomy, metacognitive regulation, and the willingness to sustain cognitive effort. Likewise, instructional design must consider the learning obstacles that may limit the development of CT, especially those arising from a lack of alignment among tasks, teaching methods, students’ developmental levels, and the epistemological complexity of the content (Akbar et al., 2025).

In higher education, this implies promoting strategies such as problem-solving, project-based learning, design-based learning, case studies, argumentative discussion, peer assessment, and collaborative learning, provided that these strategies are articulated with explicit teacher mediation, guiding questions, authentic experiences, and systematic opportunities for reflection (Abrami et al., 2015; Bezanilla et al., 2019; Ostendorf & Thoma, 2022). These strategies foster not only intellectual autonomy and metacognitive regulation, but also participation in discursive practices in which students are required to listen, justify, compare, and reformulate their ideas.

Thus, instructional mediation should be understood as a pedagogical condition that may support the activation of motivation, the regulation of metacognition, the quality of socio-linguistic interaction, and the mobilization of critical cognitive processes. Nevertheless, its effect should not be assumed as automatic. Excessively directive mediation may restrict intellectual autonomy, while poorly structured interaction may reproduce dominant positions, conformity, or the uncritical reinforcement of shared beliefs. Therefore, teacher mediation promotes CT only when it creates opportunities for autonomous inquiry, evidence-based argumentation, reflective feedback, and critical examination of assumptions.

### 3.4. Situated Critical Thinking Performance

Situated CT performance refers to the way in which critical cognitive processes, motivational engagement, metacognitive regulation, socio-linguistic interaction, and instructional mediation are articulated in authentic academic practices. In this model, CT is not reduced to an abstract individual capacity but is understood as a situated, reflective, and contextualized performance that emerges when students are required to analyse information, evaluate evidence, justify arguments, consider alternative perspectives, and regulate their own reasoning in response to meaningful problems.

Accordingly, the depth of CT achieved by students depends on the interaction between their individual resources and the pedagogical and discursive conditions that enable its development. Teacher instructional mediation creates learning opportunities; social interaction and language allow reasoning to be externalized and transformed; motivation sustains intellectual effort; metacognition regulates the process; and critical cognitive processes constitute the operational core of CT. In this way, CT is understood as a situated, reflective, and contextualized performance that emerges through authentic academic practices supported by intentional pedagogical mediation (C. J. Ossa-Cornejo & Díaz-Mujica, 2017; Davies & Barnett, 2015; Bezanilla et al., 2019; Ostendorf & Thoma, 2022).

### 3.5. Evidence-Based Decision-Making as a Formative Outcome

Evidence-based decision-making is understood as a formative outcome of situated CT. While critical cognitive processes make it possible to analyse information, evaluate evidence, construct arguments, and identify biases, situated CT performance enables students to use these processes in authentic academic, professional, and social contexts. Therefore, decision-making represents the point at which critical reasoning is expressed in justified judgments and responsible courses of action.

Consistent with Saíz and Rivas (2011, 2016), CT is not limited to abstract reasoning but is oriented toward problem solving and informed, ethical, and responsible decision-making in academic, professional, and personal contexts. From this perspective, the model proposes that critical cognitive processes support situated CT performance, and that situated CT performance, in turn, contributes to evidence-based decision-making. These two relationships should be treated as theoretically distinct propositions that require future empirical validation (Ludin, 2018).

In synthesis, the theoretical organization of the model distinguishes the cognitive core of CT from the factors and conditions that support its activation, regulation, expression, and educational development. Metacognition regulates reasoning; motivation activates and sustains intellectual effort; socio-linguistic interaction enables the externalization and reconstruction of reasoning; teacher instructional mediation organizes the conditions for its development; situated CT performance expresses the articulation of these components in authentic academic practices; and evidence-based decision-making represents the expected formative outcome.

## 4. Proposal of a Pedagogical-Interindividual Model of Critical Thinking (CT)

### 4.1. Rationale for the Integration

The proposed model is grounded in the need to understand CT in higher education as a situated, pedagogically promoted, and socially mediated performance. From this perspective, CT is conceived as a process that is activated, regulated, and deepened within specific educational contexts. To avoid an excessive expansion of the construct, the model distinguishes between constitutive processes of CT, individual factors of activation and regulation, interindividual mechanisms, and instructional-contextual conditions.

Within this framework, critical cognitive processes constitute the operational core of CT, as they enable individuals to analyse information, conduct inquiry, evaluate evidence, construct arguments, identify biases, and formulate well-founded judgments. It is important to note that the activation and quality of these processes do not occur automatically; rather, they depend on motivational factors that sustain interest, autonomy, task value, and persistence in the face of complexity, as well as on metacognitive processes that allow individuals to plan, monitor, regulate, and evaluate their own reasoning.

Social interaction and language are understood as an interindividual socio-linguistic mechanism through which critical reasoning is externalized, contrasted, and reconstructed in dialogue with others. Through language, students formulate questions, articulate reasoning, justify positions, consider counterarguments, negotiate meanings, and participate in academic discourse practices. Therefore, socio-linguistic interaction is not viewed as a stage that follows individual processes but rather as a cross-cutting mechanism capable of enhancing motivation, fostering metacognitive regulation, and enriching the quality of critical cognitive processes.

Teacher instructional mediation, in turn, constitutes an instructional-contextual condition that organizes opportunities for the development of CT in the university classroom. Strategies such as the problematization of content, higher-order questioning, formative feedback, argumentative discussion, case analysis, and authentic tasks aimed at justifying decisions create learning situations that require deep reasoning. These pedagogical conditions can activate motivation, promote metacognitive reflection, encourage argumentative interaction, and guide the mobilization of critical cognitive processes.

In this way, the model proposes a dynamic articulation among levels: instructional mediation explains the pedagogical conditions under which CT is promoted; social interaction and language explain how reasoning is expressed, challenged, and transformed; motivation explains why and with what degree of commitment intellectual effort is sustained; metacognition explains how thinking is regulated; and critical cognition explains which processes enable the construction of well-founded judgments. Accordingly, the depth of CT achieved in the university classroom depends on the interaction among individual resources, discursive practices, and pedagogical conditions that foster reflective, self-regulated, and contextualized reasoning.

Therefore, the contribution of the model lies in functionally organizing multiple factors previously studied within a pedagogical-interindividual architecture. This organization makes it possible to explain situated CT as the result of the interaction among a cognitive core, individual factors of activation and regulation, socio-linguistic mechanisms of mediation, and instructional conditions that enable its intentional development in higher education.

### 4.2. Theoretical Propositions of the Model

Based on the theoretical architecture proposed in this Hypothesis Paper, the model formulates five theoretically derived propositions intended to guide future empirical validation. These propositions should be understood as testable theoretical expectations rather than as empirically established causal mechanisms.

**P1.**  *Teacher instructional mediation and critical cognitive processes. Teacher instructional mediation is expected to be positively associated with the activation of critical cognitive processes when teaching practices include authentic problems, higher-order questions, formative feedback, argumentative discussion, and opportunities for students to justify decisions based on evidence.*

**P2.**  *Socio-linguistic and argumentative interaction as a mediating mechanism. Socio-linguistic and argumentative interaction is expected to mediate the relationship between teacher instructional mediation and the development of critical cognitive processes. Through dialogue, justification, counterargument, and the comparison of perspectives, students may externalize, examine, and reconstruct their reasoning.*

**P3.**  *Motivation and metacognition contribute to situated critical thinking through distinct but complementary intraindividual pathways.*

**P3a.**  *Motivation is expected to predict students’ willingness, engagement, and persistence in cognitively demanding tasks that require analysis, inquiry, argumentation, evidence evaluation, and bias identification.*

**P3b.**  *Metacognition is expected to support the planning, monitoring, regulation, and evaluation of reasoning, thereby contributing to the quality of critical cognitive processing.*

**P3c.**  *Metacognition may contribute indirectly to critical thinking through its positive association with motivation to think critically, particularly by strengthening students’ willingness, perceived value, and perceived usefulness of engaging in cognitively demanding tasks.*

**P4.**  *Critical cognitive processes and situated critical thinking performance. Critical cognitive processes, including analysis, inference, evaluation, argumentation, and problem solving, are expected to positively support situated critical thinking performance in authentic academic tasks.*

**P5.**  *Situated critical thinking performance and evidence-based decision-making. Situated critical thinking performance is expected to positively contribute to evidence-based decision-making, insofar as students use critical reasoning to evaluate information, justify judgments, consider alternatives, and make responsible decisions in academic, professional, and social contexts.*

## 5. Discussion and Future Directions

### 5.1. Theoretical Contribution of the Model

Although several previous frameworks have contributed to understanding CT as a complex and multidimensional construct, the novelty of the present model lies in the functional organization of these components within a pedagogical-interindividual architecture. Ossa-Cornejo and Díaz-Mujica emphasize the distinction between intraindividual and interindividual perspectives of CT, highlighting the need to understand critical thinking as both an internal cognitive process and a socially situated practice. Davies and Barnett broaden the understanding of CT by relating it to higher education purposes and to the development of criticality beyond instrumental reasoning. Similarly, the multifactorial account reported by Jaramillo Gómez et al. (2025) recognizes the relevance of cognitive, motivational, social, and contextual factors in the development of CT.

The present model builds on these contributions but adds a more explicit functional differentiation among components. Specifically, it distinguishes the cognitive core of CT from the intraindividual regulatory and activating factors, the interindividual socio-linguistic mechanism, the instructional-contextual condition, the situated performance of CT, and evidence-based decision-making as a formative outcome. In this sense, the contribution of the model is not the isolated inclusion of these components, but their organization into a theoretically testable architecture that specifies how CT may be activated, regulated, socially mediated, pedagogically supported, and expressed in situated decision-making.

Accordingly, the distinctive contribution of the model consists of explaining how CT can develop within specific university contexts: instructional mediation creates pedagogical conditions; social interaction and language transform those conditions into argumentative practices; motivation sustains intellectual engagement; metacognition regulates reasoning; and critical cognitive processes enable the construction of well-founded judgments. Thus, CT is understood not merely as an individual capacity but as a situated performance that emerges from the interaction among students’ internal resources, discursive practices, and intentionally designed pedagogical conditions.

This organization makes it possible to overcome fragmented understandings of CT, in which cognition, motivation, metacognition, language, and instruction are often addressed separately. At the same time, it avoids excessively broadening the construct by not considering all these elements as internal dimensions of critical thinking, but rather as components with differentiated functions within a relational model. Consequently, the theoretical value of the proposal lies in its delineation of a pedagogical-interindividual explanatory pathway for understanding how critical thinking is activated, regulated, and deepened in higher education.

Although the proposed model emphasizes the positive role of teacher instructional mediation and socio-linguistic interaction in the development of critical thinking, these processes should not be understood as automatically beneficial. Their contribution depends on specific pedagogical and epistemic conditions. Teacher mediation may support critical thinking when guidance is progressively adjusted, when students are encouraged to justify their reasoning, and when feedback promotes intellectual autonomy rather than dependence. Conversely, excessively directive mediation may restrict students’ opportunities to formulate questions, evaluate evidence, and construct their own judgments.

Similarly, socio-linguistic and argumentative interaction may promote critical thinking only when dialogue is oriented toward evidence-based reasoning, the comparison of perspectives, and the critical examination of assumptions. In poorly structured interactions, social exchange may reinforce conformity, dominant positions, superficial consensus, or uncritically shared beliefs. Therefore, the interindividual mechanism proposed in the model requires dialogic conditions that foster justification, counterargument, epistemic openness, and respect for alternative viewpoints.

Finally, alternative theoretical pathways should also be considered. Students with stronger critical thinking skills may elicit higher-quality dialogue, engage more effectively with teacher mediation, or participate more actively in socio-linguistic interaction. Likewise, motivation may influence students’ willingness to participate in argumentative dialogue, rather than operating only after such interaction has occurred. These possible reciprocal relationships do not invalidate the proposed model; rather, they delimit its scope and indicate directions for future empirical validation.

### 5.2. Implications for Higher Education

The pedagogical-interindividual model of critical thinking has important implications for higher education because it situates its development not only within students’ individual capacities but also within the institutional, curricular, instructional, and assessment conditions that support its enactment. Various studies warn that transmissive teaching practices, fragmented curricula, limited teacher preparation, and institutional cultures that are not open to questioning may constrain the development of CT (Aliakbari & Sadeghdaghighi, 2013; Bezanilla et al., 2021; Gharibi et al., 2024). In this regard, strengthening CT requires educators to understand its nature, educational value, and instructional strategies, given that teachers with higher levels of CT tend to demonstrate stronger pedagogical competencies for responding to contemporary educational demands (Kuloğlu & Karabekmez, 2022).

From a curricular perspective, CT should not be reduced to an implicit transversal competency or a general statement within graduate profiles. Instead, it should be integrated explicitly, progressively, and be made assessable throughout the educational trajectory (Facione, 1990; Halpern, 1997, 2014; Thompson, 2011; Ennis, 2018). This requires shifting the emphasis away from content coverage toward learning experiences that foster inquiry, evidence analysis, argumentation, problem-solving, and evidence-based decision-making within authentic academic and professional contexts (Paul & Elder, 2019; Rodríguez-Sandoval et al., 2022).

From an instructional perspective, the model calls for intentional, sequenced, and contextualized educational designs that mobilize cognitive, metacognitive, motivational, and socio-linguistic processes. Evidence suggests that strategies such as problem-based learning, project-based learning, design-based learning, case analysis, argumentative discussion, peer assessment, and collaborative learning can foster CT when combined with explicit instructional mediation, guiding questions, formative feedback, and systematic opportunities for reflection (Ostendorf & Thoma, 2022; Akbar et al., 2025). Within this framework, language and social interaction are not ancillary elements but pedagogical mechanisms that enable students to make assumptions explicit, formulate counterarguments, negotiate meanings, and construct shared knowledge.

The emergence of generative artificial intelligence (AI) introduces new opportunities and risks for the development of CT (Romero et al., 2024). Generative AI tools, including large-language-model-based chatbots, academic chatbots, automated feedback systems, and adaptive platforms can support information analysis, exploration of perspectives, question formulation, and evidence-based argumentation, particularly when integrated into active methodologies such as project-based learning, collaborative learning, the Socratic method, design thinking, or problem-solving approaches (Melisa et al., 2025; Salido et al., 2025; Aquije et al., 2025; Muñoz-Martínez et al., 2025). However, their educational potential depends on clear objectives, explicit criteria, critical digital literacy, and systematic instructional mediation. Otherwise, they may encourage uncritical acceptance of automated responses, cognitive dependency, reproduction of biases, academic integrity issues, and inequalities in access to and meaningful use of these technologies (Gerlich, 2025, Muñoz-Martínez et al., 2025; Vesga Bravo & Lancheros Bohórquez, 2025). Therefore, AI should be understood as a techno-pedagogical mediation that can either promote or constrain CT depending on the quality of instructional design and the opportunities created for questioning, verifying, and critically justifying information generated by intelligent systems (Inayati et al., 2026).

Finally, assessment practices are decisive for consolidating CT in higher education. Assessing this competency requires moving beyond memorization and content reproduction by incorporating authentic tasks that allow for the observation of analysis, inference, evidence evaluation, decision-making, metacognitive reflection, and argumentation (Saíz & Rivas, 2017). To this end, educators may employ rubrics, reflective journals, self-assessments, think-aloud protocols, academic debates, Socratic discussions, collaborative case analyses, oral defence of arguments, peer review, and argumentative writing. These strategies make it possible to evaluate not only the final product but also the cognitive, metacognitive, motivational, and socio-linguistic processes through which critical thinking is expressed, regulated, and deepened in specific educational contexts (C. J. Ossa-Cornejo et al., 2023; Valenzuela et al., 2023).

### 5.3. Directions for Empirical Validation of the Model

Future research should empirically validate the proposed pedagogical-interindividual model by analysing the relationships among teacher instructional mediation, argumentative socio-linguistic interaction, motivational factors, metacognitive regulation, and critical cognitive processes across different higher education contexts. Because these components operate at different levels, validation should not be limited to self-report instruments or conventional structural equation modelling. Longitudinal, multilevel, and intervention-based designs are needed to examine how classroom instructional conditions influence discursive interaction, motivation, metacognition, and students’ critical thinking performance.

As an initial step, it will be necessary to operationalize the constructs included in the model and examine their structure through exploration and confirmatory factor analyses, as well as their convergent validity, discriminant validity, and measurement invariance across disciplinary areas, educational levels, instructional modalities, and institutional contexts. Subsequently, direct, indirect, mediating, and moderating relationships could be examined through multilevel or longitudinal models. Furthermore, self-report measures should be complemented with performance-based tasks, case analyses, classroom observations, analyses of argumentative discourse, critical reasoning rubrics, and behavioural indicators of participation, regulation, and decision-making.

This line of validation would make it possible to determine the explanatory power of the model, specify the conditions under which it can be applied, and evaluate its usefulness in guiding curriculum design, instructional mediation, authentic assessment, and pedagogical innovation aimed at strengthening situated critical thinking in higher education.

### 5.4. Limitations

The proposed model has several limitations that should be considered when interpreting its scope. First, the conceptual review followed an integrative rather than systematic procedure and therefore does not ensure exhaustive coverage of the literature, which may introduce selection bias. Second, the empirical foundation is uneven across the components of the model, particularly regarding the specific cross-level relationships proposed among instructional mediation, socio-linguistic interaction, motivation, metacognition, and critical cognitive processes. Third, part of the evidence used to contextualize the proposal derives from Latin American and Chilean higher education, while other components draw on broader international literature; consequently, the generalizability of the proposed architecture across educational systems remains to be established. Fourth, although the distinction between constitutive, regulatory, activating, interindividual, and instructional-contextual components improves conceptual delimitation, these functional boundaries require empirical testing. Finally, the model has not yet been validated longitudinally or across disciplines and institutions; therefore, its causal, mediating, moderating, and reciprocal relationships should be interpreted as theoretical expectations rather than established effects.

## 6. Conclusions

The present theoretical study supports the view that CT constitutes a complex phenomenon whose understanding extends beyond approaches focused exclusively on cognitive skills. Although analysis, evaluation, inference, argumentation, and evidence-based decision-making continue to represent the operational core of CT, the evidence reviewed indicates that its activation, regulation, and deepening also depend on metacognitive, motivational, socio-linguistic, and instructional factors that operate at different levels of the educational process.

In this context, the principal contribution of the article is the proposal of a pedagogical-interindividual model of situated critical thinking in higher education. Unlike conceptions that group all components as internal dimensions of CT, the model distinguishes specific functions: critical cognitive processes constitute the operational core of the construct; metacognition acts as a regulatory system for reasoning; motivation functions as an activating and sustaining condition for deep thinking; social interaction and language operate as a socio-linguistic mechanism that enables the externalization, comparison, and reconstruction of reasoning; and teacher instructional mediation is conceived as an instructional-contextual condition that organizes opportunities for its development within the university classroom.

This distinction makes it possible to integrate the intraindividual and interindividual perspectives of CT. From the intraindividual perspective, the model recognizes the importance of cognitive skills, self-regulation, and students’ motivational disposition. From the interindividual perspective, it emphasizes that critical thinking develops through academic practices mediated by language, argumentation, dialogue, interaction with others, and the pedagogical conditions created by instructors. Consequently, CT is not reduced to an individual capacity but is understood as a situated, reflective, and socially mediated performance.

The implications of the model are relevant for both research and educational practice. From a research perspective, the proposal offers a conceptual framework that is amenable to empirical validation through designs that consider the multilevel nature of the phenomenon, incorporating not only self-report instruments and structural equation models but also longitudinal studies, intervention designs, classroom observation, performance tasks, and analyses of argumentative discourse. From an educational perspective, the model guides curriculum design, instructional mediation, and assessment toward learning experiences that promote inquiry, argumentation, metacognitive reflection, motivation for deep thinking, and evidence-based decision-making.

Finally, the model is advanced as a testable theoretical hypothesis rather than as a framework whose internal relationships have already been empirically established. Its validation will require research across diverse disciplinary, institutional, and instructional contexts to examine the proposed direct and indirect relationships among teacher instructional mediation, argumentative socio-linguistic interaction, motivation, metacognitive regulation, critical cognitive processes, situated critical thinking performance, and evidence-based decision-making. Future studies should also explore possible reciprocal or moderating relationships that may emerge among these components. Such research will help clarify the relative contribution and boundary conditions of each component and determine the extent to which the proposed functional architecture explains how critical thinking is activated, regulated, expressed, and deepened in higher education.

## Data Availability

No new empirical data were generated or analyzed in this theoretical/conceptual study. The original contributions presented in the article are included in the article. Further inquiries can be directed to the corresponding author.
